# The “Polo Cassia” project: a mental health service for integrated treatments of co-occurring disorders in adolescents and young adults

**DOI:** 10.1192/j.eurpsy.2025.1651

**Published:** 2025-08-26

**Authors:** S. Navari, R. Carpentieri, S. Ceccucci, A. Picello, F. Di Nicola, L. D’Agostino, D. Giovannetti, D. DeLuca, L. Ferrara, C. Riitano, M. L. Carenti, A. Correale, S. D’Andrea, L. Marcellini, S. Oprea, F. Summa, A. Demaggi, I. Zappino, M. R. Barbera, V. Trincia, G. Ducci

**Affiliations:** 1 Dipartimento di Salute Mentale, ASL Roma1, Roma; 2 Dipartimento di Salute Mentale, ASL Roma, Rome, Italy

## Abstract

**Introduction:**

The term “co-occurring disorders” (COD) refers to the coexistence of two or more psychiatric disorders especially substance use disorder (SUD) and other psychiatric disorders. In particular, the prevalence of COD increases globally and is linked to a higher risk of worse outcome. Furthermore, especially in adolescence, the goal is to provide an early and proper answer both in terms of care and prevention. On the opposite, there is a lack of integrated management strategies requiring an effective collaboration of different mental health disciplines in order to receive appropriate care. Although European countries have established mental health policies, a lack of comprehensive and structured mental health intervention programs remains.To answer these needs we developed an Integrated Mental Health Service for patients with co-occurring disorders aged up to 30 years, lining varied challenges, including both the interception of patient’s needs and the practical organization of interventions.

**Objectives:**

This study aims to identify the impact of an Integrated Mental Health Service, “Polo Cassia”, coordinated by a team of professionals based in the same physical location, on the outcome of co-occurring disorders in patients aged up to 30 years at their first contact with mental health services. Furthermore, we aim to identify which transversal clinical dimensions and risk factors might benefit from an early integrated and tailored treatment.

**Methods:**

The project is carried out at the Community Mental Health Center (CMHC) of District 15th, ASL Rome 1. The final study sample will include all the patients referred in one year to “Polo Cassia”. Clinical and socio-demographic parameters are collected in details, Global Assessment Functioning (GAF), and Quality of Life Enjoyment and Satisfaction Questionnaire (Q-LES-Q) are administered at baseline (To), after 6 months (T1) and 1 year (T2).

**Results:**

Among the study sample referred to Polo Cassia (53% male, mean age 20,33 ±4,47), 50% of the patients comes directly from a first triage evaluation, 35% from other mental health services and 15% from other health services (e.g. diabetes centre). The 60% of the patients have SUD coexisting with other psychiatric disorders. From updated results, we expect to be able to verify the impact of an early integrated treatment on global function and quality of life in our sample of patients. Furthermore, we expect to characterize the transversal clinical dimensions which will actually respond to an early integrated treatment with positive clinical outcomes and subjective satisfaction.

**Conclusions:**

The experience of structured integrated models of care for co-occurring psychiatric disorders is necessary to inform the future development of Mental Health Services.

**Disclosure of Interest:**

None Declared

